# Making Life Better for Older Adults and Families: My Journey in Intervention Science

**DOI:** 10.1093/geroni/igaf122.336

**Published:** 2025-12-31

**Authors:** Laura Gitlin

**Affiliations:** Drexel University, Philadelphia, Pennsylvania, United States

## Abstract

Intervention science is a dynamic and challenging field that addresses critical public health issues by bridging the gap between theory and practice. It focuses on designing, implementing, and evaluating novel approaches that have the potential to significantly improve the health and well-being of individuals, families, and communities. My research journey in this area has been driven by a deep commitment to understanding how behavioral, psychological, and environmental factors intersect and can be modified, even for individuals facing serious chronic illnesses. Over my 40+ year career, I have had the privilege of developing and testing numerous interventions, in collaboration with various teams, which have been shown through clinical trials to be efficacious, acceptable, feasible, and have cost-savings. Many of these interventions have been adopted worldwide and adapted for different delivery contexts. Using COPE and similar programs as exemplars, I will trace my journey through intervention science—from the initial inception of ideas to their implementation. This includes the transition from developing and testing face-to-face, home-based intensive programs to innovative, technology-driven digital solutions. While intervention science holds great promise, significant gaps remain between the generation of effective interventions and their integration into real-world settings. These knowledge-to-practice gaps continue to restrict access to evidence-based practices for many families, posing a challenge to the field and driving my career decisions. This talk will explore the personal, ethical, and professional dilemmas encountered on the journey of an intervention scientist, offering insights into how we can bridge these gaps and improve the reach and impact of interventions.

